# A structural model of the immune checkpoint CD160–HVEM complex derived from HDX-mass spectrometry and molecular modeling

**DOI:** 10.18632/oncotarget.26570

**Published:** 2019-01-11

**Authors:** Katarzyna Kuncewicz, Marta Spodzieja, Adam Sieradzan, Agnieszka Karczyńska, Katarzyna Dąbrowska, Michał Dadlez, Daniel E. Speiser, Laurent Derre, Sylwia Rodziewicz-Motowidło

**Affiliations:** ^1^ University of Gdansk, Faculty of Chemistry, Department of Biomedical Chemistry, Gdansk, Poland; ^2^ University of Gdansk, Faculty of Chemistry, Department of Theoretical Chemistry, Gdansk, Poland; ^3^ Polish Academy of Sciences, Institute of Biochemistry and Biophysics, Mass Spectrometry Laboratory, Warsaw, Poland; ^4^ Ludwig Cancer Research Center, Department of Oncology, Epalinges, Switzerland; ^5^ University Hospital of Lausanne (CHUV), Urology Department, Urology Research Unit, Lausanne, Switzerland

**Keywords:** CD160, HVEM, immune checkpoints, hydrogen/deuterium exchange combined to mass spectrometry, molecular modeling

## Abstract

CD160 is a T cell coinhibitory molecule that interacts with the herpes virus entry mediator (HVEM) on antigen-presenting cells to provide an inhibitory signal to T cells. To date, the structure of CD160 and its complex with HVEM are unknown. Here, we have identified the fragments of CD160 interacting with HVEM using ELISA tests, hydrogen/deuterium studies, affinity chromatography and mass spectrometry (MS). By combining hydrogen/deuterium exchange and mass spectrometry (HDX-MS) we obtained key information about the tertiary structure of CD160, predicting the 3D structure of the CD160–HVEM complex. Our results provide insights into the molecular architecture of this complex, serving as a useful basis for designing inhibitors for future immunotherapies.

## INTRODUCTION

Among the most promising approaches to activate the immune system is the inhibition or stimulation of immune checkpoints. Cosignaling molecules have been implicated in the dysregulation of immune responses in autoimmunity, chronic infections, and cancers [1–3]. Numerous immune checkpoints (either coinhibitory or costimulatory) have been identified, which are candidate targets for immunotherapy: CTLA-4 and its ligands B7.1/B7.2 [4, 5], PD-1 and PDL-1/PDL-2 [6, 7], TIGIT and CD155 [8], OX40 and OX40L [9], TIM-3 and Galectin-9 [10], and the recently discovered BTLA and HVEM [11, 12] or CD160 and HVEM [13]. Most cosignaling molecules are members of the immunoglobulin superfamily (IgSF) or tumor necrosis factor superfamily (TNFSF). In general, the Ig superfamily receptors bind to the Ig superfamily ligands and TNF receptors superfamily (TNFRSF) bind to TNF ligands superfamily (TNFSF). Until now, the CD160–HVEM and BTLA–HVEM interactions are the only examples of signaling complexes between members of the two distinct families: IgSF and TNFRSF.

Herpes virus entry mediator (HVEM) acts both as a ligand for the Ig superfamily proteins, BTLA [11, 12], and CD160 [13] and as a receptor for TNFSF ligands, LIGHT, and Lymphotoxin α (LTα) [14, 15]. Interaction of HVEM with CD160 and BTLA triggers a coinhibitory signal to T-cell activation, whereas its interaction with LTα and LIGHT leads to a costimulatory signal. Thus, HVEM could regulate the host immune response, and that process depends on the type of ligand of HVEM that is involved [16, 17].

HVEM is a transmembrane protein, which has three full cysteine-rich domains (CRD), each of them stabilized by three disulfide bridges. The fourth CRD domain of HVEM contains only two of the three disulfide bonds [18–20]. The analysis of binding sites of HVEM showed that LIGHT and LTα interact with the CRD2 and CRD3 domains of HVEM [21, 22], while BTLA [21] and CD160 [13] bind to the CRD1 region.

In contrast to BTLA and HVEM [21], no crystal structure of the CD160–HVEM complex is available. The CRD1 domain of HVEM, crucial for the binding of coinhibitory molecules, interacts also with the glycoprotein D (gD) from the herpes simplex virus [23–25]. The interactions in the BTLA–HVEM and gD–HVEM complexes are very similar. Both proteins bind to HVEM using two short β-strand fragments, which form hydrogen bonds with β-strand presented in the CRD1 domain of HVEM. Despite the similarity of the structure of these strands in BTLA and gD, a comparison of their sequences shows that they have no sequence identity and only have modest sequence similarity, indicating the dominant role of backbone hydrogen bonds in the intermolecular β-sheet of the complex formation [21, 26]. Some studies showed that HVEM probably interacts with CD160 in a similar way as BTLA and gD. CD160 binds to HVEM with a similar affinity, but at a slower dissociation rate than BTLA and competes with BTLA for binding to HVEM [27].

CD160 is a glycosylphosphatidylinositol-anchored membrane glycoprotein (CD160–GPI) with a single IgV-like domain that belongs to the Ig superfamily. Three other isoforms of CD160 are also described (CD160DIg–GPI, CD160–TM, CD160DIg–TM), which are generated by alternative splicing. They differ from each other by the presence or absence of the Ig domain, the transmembrane domain, and/or the GPI motif [28, 29]. CD160–GPI and CD160–TM isoforms interact with HVEM, indicating that the Ig domain is necessary for the protein binding [28, 29]. Four different isoforms of CD160 have been identified, but only two of them (with IgV-like domain) can bind to HVEM [29, 30]. The extracellular domain of CD160 contains five cysteine residues, four of them form disulfide bridges (Cys18–Cys86 and Cys35–Cys42) and one in the position 87 has a free sulfhydryl group [28]. The protein also has two sites for *N*-linked glycosylation, and the asparagine in positions 2 and 111. CD160 also binds to MHC class I molecules with low affinity and this binding might enhance NK and T-cell cytolytic activity [30]. The function of CD160 has not yet been fully characterized beyond the finding that CD160 can decrease the proliferation and the cytokine production of CD4^+^ and CD8+ T-cells upon binding to HVEM [13, 29].

Herein, by combining *in vitro* and *in silico* analyses, we identified the binding sites of CD160 that are responsible for the interaction with HVEM. In addition, by performing a molecular docking of CD160 to HVEM, we generated a predictive model of the 3D structure of this complex.

## RESULTS

### H/D exchange pattern in the CD160 protein

To obtain the structural information about the CD160 protein, a HDX-MS experiment was performed. In this experiment proteins in aqueous buffer are diluted in D_2_O buffer, which results in replacement of proteins hydrogens with deuteria. The rate of exchange depends on chemical properties of amino acid residues, pH of buffer and temperature, but also on structural properties of the protein. Hydrogens involved in hydrogen bond formation do not until the H-bond is broken. High level of exchange characterizes unstructured, more dynamic regions of protein. Low levels of exchange are characteristic for rigid structures. Using mass spectrometry we can measure the mass difference resulting from hydrogen/deuterium exchange in different peptides derived from a protein after enzymatic fragmentation. It allows investigation of protein's dynamics and dynamical changes occurring as a result of interaction with other proteins, ligands, lipids etc.

**Figure 1:**
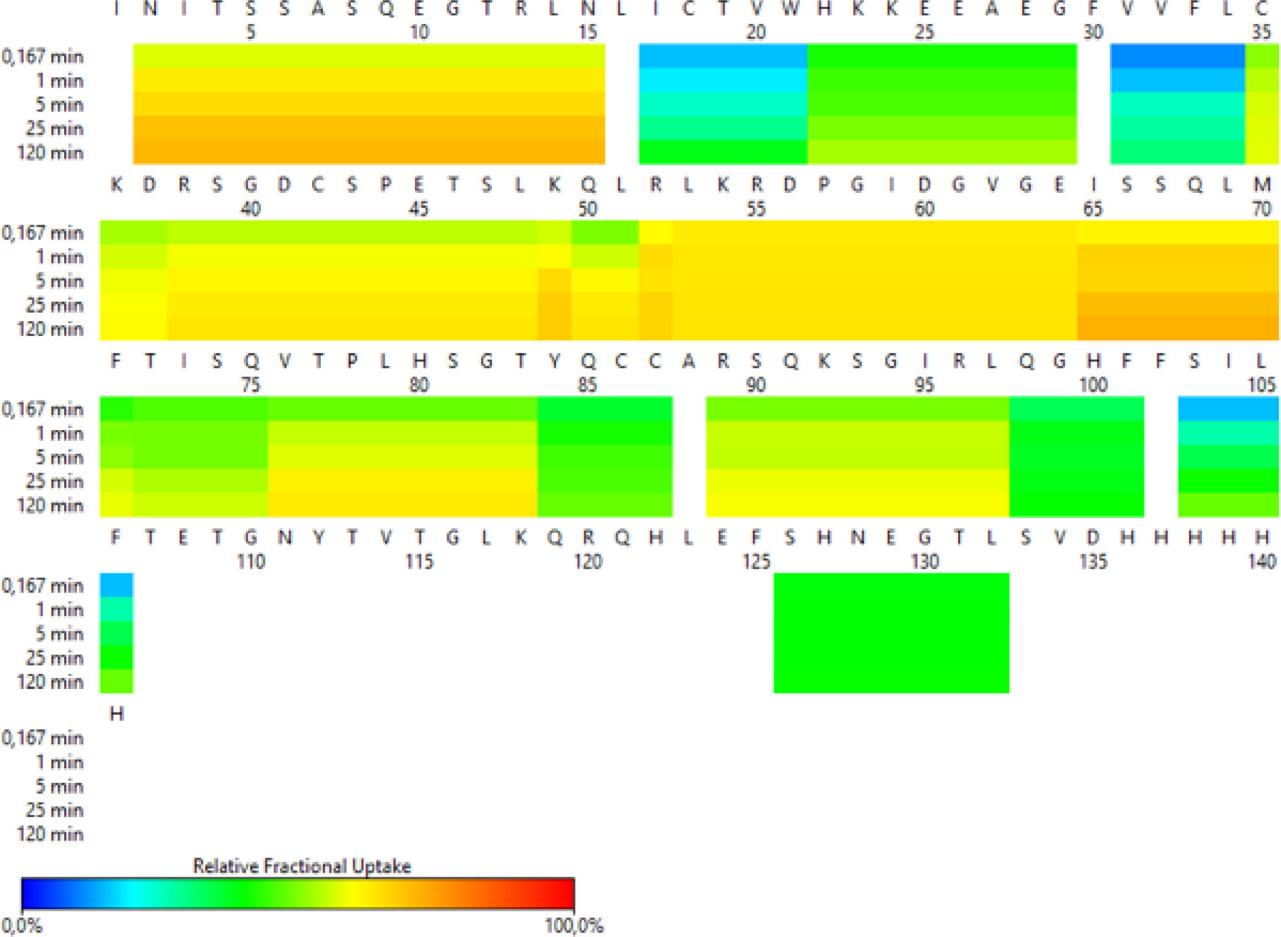
HDX-MS heat map representing the pattern of exchange in CD160 protein

In Figure 1 red and orange regions indicate CD160 protein exposed to solvent, blue and green regions are less susceptible to H/D exchange. Our results show that the H/D exchange in *N*-terminus of the CD160 protein (residues 1–15) is very fast (more than 70%), indicating a flexible structure in this region. A similar process was observed for the middle part of protein (position 35–65, 76–83 and 89–97). Residues 17–21, 31–34, 71, 83–87, 98–106 and 126–132 represent more structured regions (Figure 1).

In addition, to obtain the maximal resolution of the HDX-MS experiment and sequence coverage, the CD160 protein was digested with two enzymes (protease type XIII from *A. saitoi* and pepsin). Digestion of protein generated 45 peptides, covering sequence in 80.9% with average redundancy 5.28 (Supplementary Figure 1). The enzymatic digestion of the CD160 protein generated only one peptide ((15 amino acids (aa)) from the *N-*terminus. On the other hand, many peptide fragments were found (6 to 32 aa) from the central part of the protein. The *C*-terminal fragments (residues from 107 to 124 and from 133 to 141) of the CD160 protein were not detected in any of the performed MS experiments. The lack of *C*-terminal peptides and the presence of only one peptide from *N*-terminal part of the protein in digest mixture might be the result of glycosylation of Asn2 and Asn111 residues. The presence of carbohydrates may create a hindrance in the enzymatic digestion and ionization of proteins [31]. Furthermore, ELISA test for CD160 and deglycosylated CD160 (obtained by using PNGase F enzyme) proteins were performed. Our results indicated that the deglycosylated CD160 protein has a very low affinity to the HVEM protein. This suggests that deglycosylation changed the structure or/and activity of CD160 (Supplementary Figure 2).

### Identification of sites that help binding of CD160 to HVEM using HDX-MS

**Figure 2:**
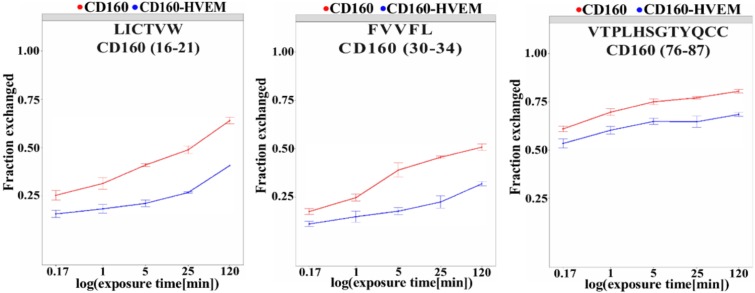
Regions of CD160 protein with important changes in deuteration level upon interaction with HVEM CD160 in complex with HVEM (blue line) and free CD160 (red line). Deuteration was measured after incubating in deuterated PBS for 10 s, 1 min, 5 min, 25 min, and 2 h, which is shown on a logarithmic scale.

Measuring the level of H/D exchange for CD160–HVEM complex and comparing the level of exchange in CD160 free protein indicates the regions, whose dynamics changed upon interaction. Figure 2 illustrates the regions that are more protected from exchange and had less H/D exchange upon protein binding are implicated in the interaction with HVEM. Deuteration was measured after incubating in deuterated PBS for 10s, 1 min, 5 min, 25 min, and 2 h. Results show that significant decrease in the deuteration of CD160–HVEM complex, as compared to CD160 alone were observed in following peptides, namely: CD160(16–21), CD160(30–34), and CD160(76–87) (Figure 2). For H/D data presenting the exchange in the remaining regions and *p*-values calculated using Student's *t*-test for peptides after n time of HD exchange see Supplementary Materials (Supplementary Figure 3 and Supplementary Table 1).

### Identification of the CD160 binding sites to HVEM using affinity chromatography and ELISA tests

Next, we synthesized 10 long peptides (20–25 (aa) long, overlapped by 8 aa) covering the Ig-V domain of CD160. In order to prevent the formation of inter- and intramolecular disulfide bridges, all cysteines were replaced by 2-aminobutyric acid (Abu), which is isosteric to cysteine. Then, peptides were analyzed in an affinity test using the microcolumn containing immobilized HVEM protein. Three fractions, the supernatant, last wash, and elutions were analyzed using mass spectrometry. In the supernatant fraction, the signal m/z corresponding to the unbound peptide was observed. In the last wash fraction, no signal was observed, which confirms that the excess of peptide was removed. The observation of m/z signal in the elution indicated that the complex between HVEM and CD160 peptide was formed. Indeed, peptides CD160(13–32), CD160(25–44), CD160(61–80), CD160(73–92) and CD160(85–104) were bound to HVEM protein (Table 1). Fragment CD160(97–116) showed very bad solubility in all the tested solutions (H_2_O, DMF, DMSO, etc.) and thus could not be analyzed using the affinity chromatography. Therefore, two additional, shorter fragments were synthetized and analyzed: CD160(97–110) and CD160(103–116) (see Table 1—marked with an asterisk (^*^)). The affinity test indicated that the peptide CD160(97–110) interacts with HVEM protein.

**Table 1:** Microcolumn affinity tests

Peptide	Sequence	[M+H]^+^_calc._	Supernatant [M+H]^+^	Last wash	Elution [M+H]^+^
**CD160 (1–20)**	INITSSASQEGTRLNLIAbuTV-NH_2_	2101.36	2101.02	n.o.	n.o.
**CD160 (13–32)**	Ac-RLNLIAbuTVWHKKEEAEGFVV-NH_2_	2394.77	2394.37	n.o.	2394.32
**CD160 (25–44)**	Ac-EEAEGFVVFLAbuKDRSGDAbuSP-NH_2_	2193.37	2193.21	n.o.	2193.19
**CD160 (37–56)**	Ac-DRSGDAbuSPETSLKQLRLKRD-NH_2_	2327.56	2327.17	n.o.	n.o.
**CD160 (49–68)**	Ac-KQLRLKRDPGIDGVGEISSQ-NH_2_	2237.52	2237.10	n.o.	n.o.
**CD160 (61–80)**	Ac-GVGEISSQLMFTISQVTPLH-NH_2_	2185.50	2185.13	n.o.	2185.23
**CD160 (73–92)**	Ac-ISQVTPLHSGTYQAbuAbuARSQK-NH_2_	2212.47	2210.11	n.o.	2210.19
**CD160 (85–104)**	Ac-QAbuAbuARSQKSGIRLQGHFFSI-NH_2_	2271.58	2271.43	n.o.	2271.15
**CD160 (97–116)**	Ac-LQGHFFSILFTETGNYTVTG-NH_2_	2273.50	-	-	-
^*^CD160(97-110)	Ac-LQGHFFSILFTETG-NH_2_	1637.83	1637.65	n.o.	1637.65
^*^CD160(103-116)	Ac-SILFTETGNYTVTG-NH_2_	1543.67	1565.60 [M+Na]+	n.o.	n.o.
**CD160(109-133)**	Ac-TGNYTVTGLKQRQHLEFSHNEGTLS-NH_2_	2859.07	2859.23	n.o.	n.o.

n.o. – not observed; - – not results.

**Figure 3:**
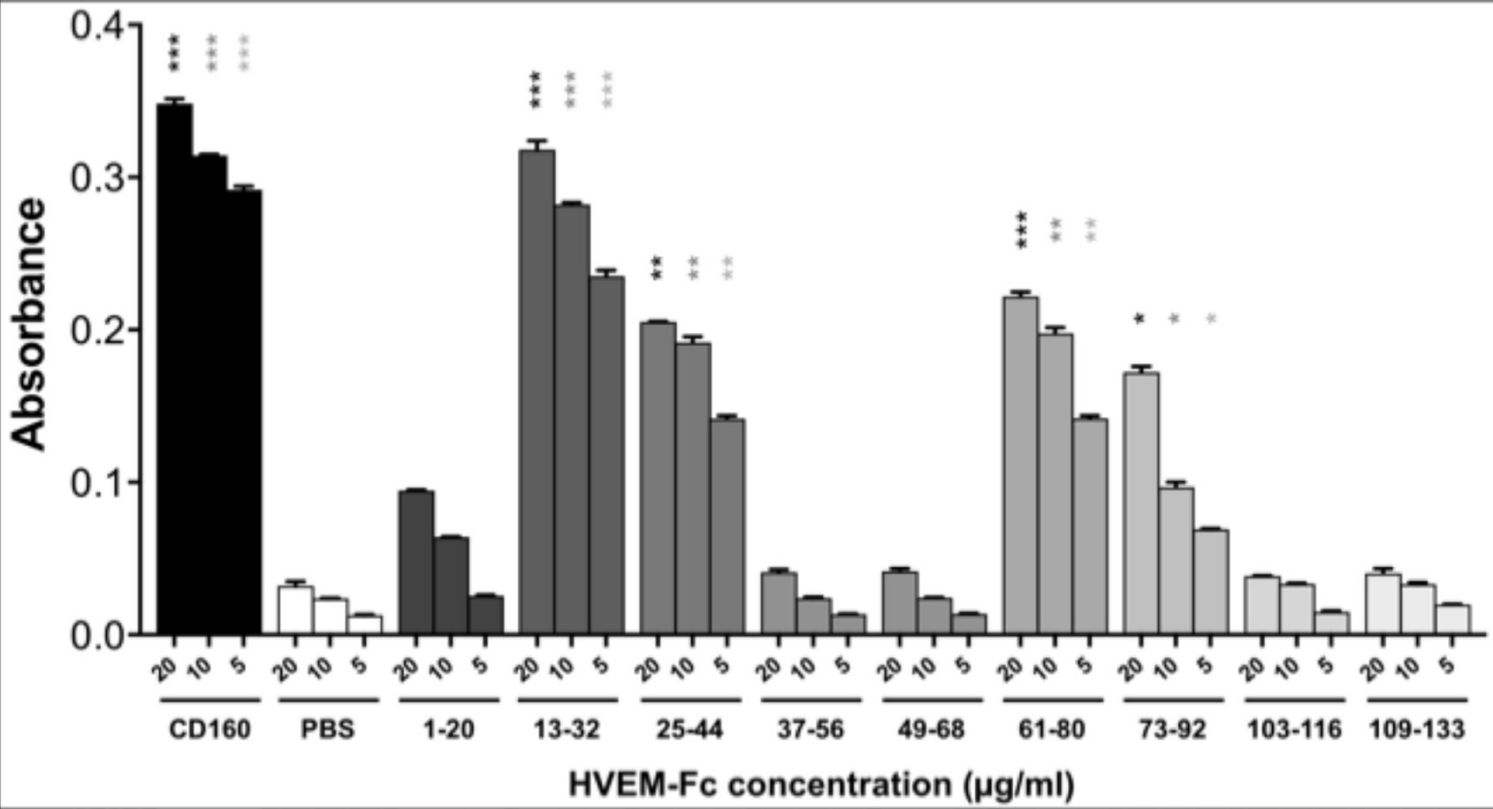
ELISA test results for the binding of CD160 protein and its fragments to HVEM-Fc protein Statistical analysis was performed by comparing each concentration of HVEM-Fc to the same concentration in the PBS condition.^***^p < 0.001, ^**^p < 0.01, ^*^p < 0.05.

Then, in order to determine which of the above CD160 fragments has higher affinity to HVEM, an ELISA test was performed. For this purpose, CD160 protein was biotinylated, whereas CD160 fragments were elongated by five glycine residue and biotin to attach it on a streptavidin-coated surface. In Figure 3 the absorbance for the HVEM-Fc in different concentrations (20, 10, and 5 μg/ml) is shown. The ELISA results show that the strongest affinity to the HVEM revealed CD160(13–32) peptide. We found that three fragments - CD160(25–44), CD160(61–80), and CD160(73–92) interact also with HVEM, but with a lower affinity. The other analyzed peptides show very weak or no affinity to HVEM. Based on these results it is possible to conclude that 13–44 and 61–92 regions of CD160 can bind to HVEM protein.

For the fragments CD160(85–104) and CD160(97–110) the very strong signals in ELISA assays were observed. As negative control, we have checked what will be the absorbance signal for all peptides in the ELISA test, but without the HVEM protein. In this test the high absorbance was detected only for those two peptides - CD160(85–104) and CD160(97–110). Many assay conditions, such as various microplates, buffers, blocking solutions, concentrations, and plate-wash steps were tested by us. In each tested trial, the absorbance signal from only CD160 (85–104) and CD160 (97–110) peptides was similarly high. In order to check sequence specificity, for both interacting peptides also the scrambled peptides were synthesized and studied. Obtained results for scrambled peptides were very similar to CD160(85–104) and (97–110) peptides, what might suggest non-specific interactions of these peptides with HVEM protein (see Supplementary Figure 4).

### Structure determination of the CD160 protein

To obtain a reliable full-length structural model of CD160 protein, the I-TASSER homology modeling was established at the first step of the CD160 protein structure determination. The following protein structures from PDB, all sharing an immunoglobulin-like fold, were chosen by I-TASSER as templates in the modeling: 1TJH, 1Q8M, 4WEB, 2AGJ, 2IQA, 7FAB, 1KB9 [32]. Best homology modeling structure was obtained with high C-score −2.65. In the I-TASSER program no sequences that were homologous for the *C*-terminal part of the CD160 protein were found. Therefore, *C*-terminus fragment (119–159) was calculated in a coarse-grained force field.

Next, the final I-TASSER model (with the highest C-score) together with *C*-terminus (119–159 fragments) was selected for the structure calculation in a coarse-grained force field. To get folded structures of the CD160 protein, the multiplexed replica exchange molecular dynamics (MREMD) was performed. Finally, we obtained 20 structures (model I to XX, Supplementary Figures 5 and 6) of the CD160 protein. From all the obtained 3D structures the best one (model XX), which matched the experimental criteria (HDX-MS results, disulfide bonds positions and IgV immunoglobulin fold), was chosen as the final structure. This structure was further used for the model validation process.

**Figure 4:**
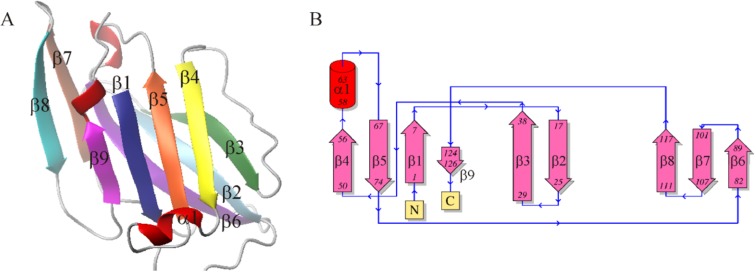
(**A**) 3D-structure of UNRES model of the CD160 protein; (**B**) Schematic topology of the CD160 protein.

The best 3D structure of the CD160 protein (Figure 4A) was then validated. The overall model quality (z-score) for the CD160 protein is −3.7 [33]. This value indicates that this model is of good quality (see Supplementary Figure 7). The final structure of the CD160 protein is a typical β-sandwich Ig fold containing nine β-strands [34]. Similarly, to other Ig-like proteins, it belongs to the IgV-like domain of the Ig superfamily and consists of two tightly packed β-sheets - one composed of 4 strands (1, 4, 5, and 9) and the other of 5 strands (2, 3, 6, 7, and 8), which are connected by nine loops (Figure 4) with one short α-helix between β4 and β5 strands. In the first and second β-sheets, all strands are arranged in an antiparallel fashion. From our experimental studies, it is possible to define that in the final structure the β2 (residues 17–28) and β3 (residues 29–37) strands and the loop between the β5 and β6 strands (residues 74–82) together form the binding site for HVEM protein.

### The CD160–HVEM complex model

**Figure 5:**
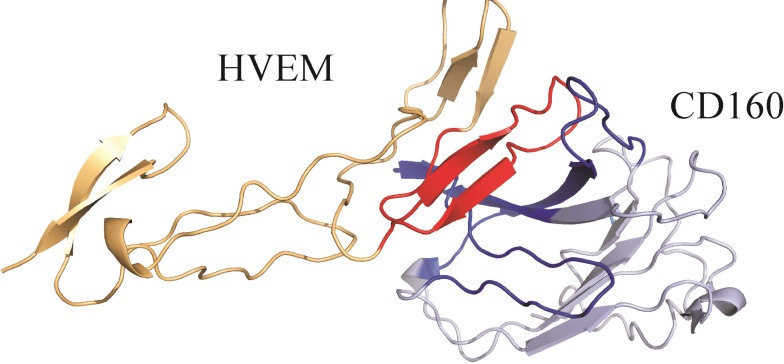
The HVEM (orange)–CD160 (light blue) complex obtained from the UNRES simulation The interacting fragments are colored in red and dark blue.

**Figure 6:**
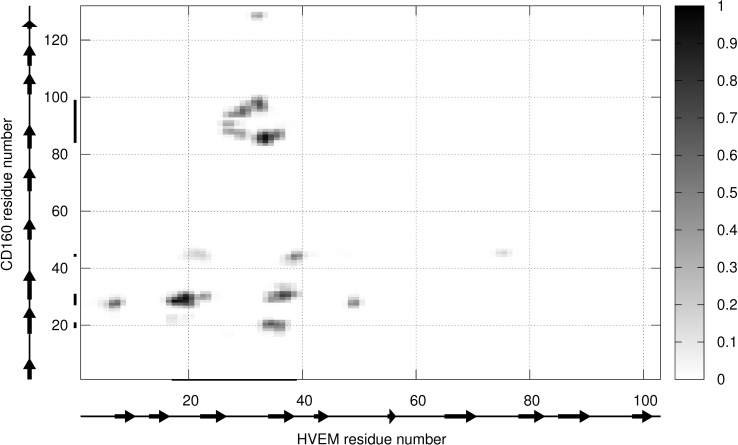
The contact map scoring obtained for CD160 and HVEM interface from coarse-grained simulations for all structures from the most probable cluster The gray scale (i.e., contact score) indicates the highest probability of contact for black and the smallest for white color.

We next formed the CD160–HVEM complex model. The predicted CD160 protein was docked to the crystal structure of HVEM (PDB code: 2AW2). Validation of UNRES docking method with use of BTLA-HVEM complex was performed, starting from random orientation of both proteins with respect to each other. UNRES docking was able to find correct (as in crystal structure) protein orientation with an average RMSD of the third cluster of 1.68 Å (see Supplementary Figure 8). The CD160–HVEM complex structure, obtained from clustering after the UNRES simulation, with the highest probability (the lowest free energy) is shown in Figure 5. In the HVEM (orange) - CD160 (light blue) complex the interacting fragments are colored in red and dark blue, respectively. The CD160 binds to HVEM by large β-sheet surface. When the contact map (colored in grayscale) between two proteins in this theoretical complex was analyzed (Figure 6), the CD160 regions 20–45 (β2 and β3 strands) and 82–100 (β6-strand and the loop between β6 and β7 strands) were found to have the highest number of contacts in the interface formation of HVEM–CD160 binding.

Later, all-atom simulation was performed, which revealed that CD160–HVEM complex is very stable as RMSD do not increase significantly (Supplementary Figure 9). The structure of the complex was stabilized after less than 1 ns of MD simulation. In order to find which fragments of both proteins are the most flexible, we have measured the backbone fluctuations along the MD trajectory. The highest fluctuations appear in the loops and terminal fragments (see Supplementary Figure 10). The lowest fluctuations could be observed for the binding fragments in both proteins. These results show that the interacting fragments are well matched. The slight increase in RMSD results from UNRES inaccuracies in structural details; however, the same residues as in the case of coarse-grained force field are involved. Nevertheless, the structure of the UNRES-predicted complex is similar to obtained after MD in AMBER (RMSD αC = 3.38 Å).

**Figure 7:**
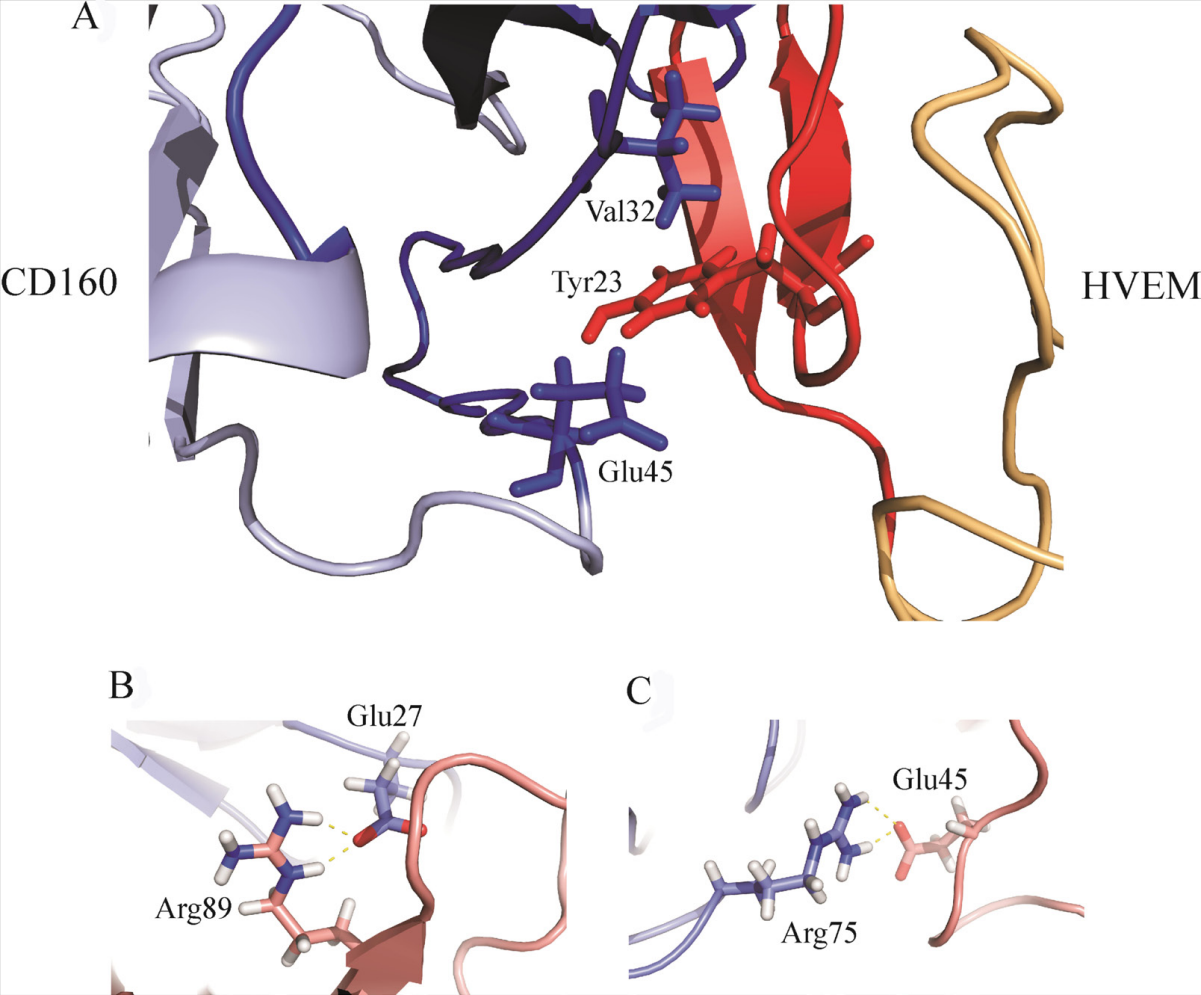
(**A**) The HVEM key residue Tyr23 interacting with Val32 and Glu45 of the CD160 protein. The colors in this drawing are the same as in Figure 5; (**B**) The visualization of salt bridge between Glu27 of HVEM (violet) and Arg89 of CD160 (pink) (**C**) The visualization of salt bridge between Arg75 of HVEM (violet) and Glu45 of CD160 (pink).

The amino acid residues 20, 22, 30, 32, 43, 44, 45, 81, 89, and 99 of the CD160 protein are involved in the binding site formation. These residues stabilize the structure of the complex by forming stable salt bridges (Glu27 of HVEM (violet) and Arg89 of CD160 (pink), Arg75 of HVEM (violet) and Glu45 of CD160 (pink); Figure 7B and 7C, respectively) and hydrophobic interactions (see Supplementary Table 2) during MD trajectory. The contribution of salt bridges and hydrophobic interactions between CD160 and HVEM proteins play a very important role in the complex stabilization of investigated proteins. Also, very important interactions were observed for Glu45^CD160^, Phe30^CD160^, and Val32^CD160^ amino acids, which are engaged in the interaction with the key Tyr23^HVEM^ [21] amino acid residue (Figure 7A).

## DISCUSSION

CD160 inhibits TCR-mediated signaling through interaction with HVEM [13], leading to the decrease of cytokine production and proliferation by T cells [29]. Moreover, CD160 may be implicated in immune regulation in different types of disease, such as chronic viral infections (HIV, HCV) [35, 36], autoimmune diseases (e.g., psoriasis) [37], and cancers [27, 38]. The current knowledge suggests that CD160 may be an interesting target for therapeutic modulation of immune responses.

In this study, using *in vitro* experiments and *in silico* modeling, we determined the binding sites of CD160 that are responsible for the interaction with HVEM, and we propose a theoretical structure of CD160 and the CD160–HVEM complex.

The 3D structure of the CD160 protein is very similar to the other protein interactions with HVEM such as BTLA and also to other Ig superfamily proteins. It is composed of two flat β-sheets, which are formed by strands β1, β4, β5, and β9 in one sheet and strands β2, β3, β6, β7, and β8 in the other (Figure 4A). So far, the crystal structures of the CD160 and the CD160–HVEM complex were not determined.

We aimed at finding the fragment of the CD160 protein targeting HVEM by using different physicochemical methods like HDX-MS, affinity test, and ELISA test. Each of these methods has their own limitations. In HDX-MS studies the protein–protein complex is created in the solution and the experiment is performed in native conditions, but this method can provide information about binding sites in protein–protein complexes only when the interaction results in allosteric changes in the surrounding structure and dynamics of proteins [39]. In affinity and ELISA tests, the protein (or peptide) is immobilized on solid phase (microcolumn or plate), which can influence the conformation of molecules [40]. Moreover, ELISA and affinity tests may give false-positive results due to nonspecific binding of peptides, proteins, or antibodies to the surface of microplates [41] or to the microcolumns. Therefore, it is reasonable to use more than one method to explore the protein interactions.

In HDX-MS studies, we analyzed the differences in deuteration level for the free CD160 and the CD160–HVEM complex. CD160 (16–21), CD160 (30–34) and CD160 (76–87) fragments are the shortest region for which significant changes were observed. This suggests that the binding sites in CD160 protein are discontinuous (formed by different fragments of CD160 sequence) and that CD160 interacts with HVEM via short (5–12 aa) fragments.

Next, the results from affinity and ELISA tests were found to be similar. Peptides which were observed in the elution fraction in affinity tests also bound to the HVEM protein in ELISA tests. The strongest interaction with HVEM (similar as the whole CD160 protein) was observed for the longest peptide CD160 (13–32), which contains the short fragment CD160 (16–21) that showed difference in the deuteration level between free and bound protein in the HDX-MS experiment. Additionally, the UNRES-derived structure of CD160–HVEM complex showed that Val20^CD160^ and His22^CD160^ are important for the protein interactions. All our data indicate that CD160 (13–32) fragment of the CD160 protein is important for the CD160–HVEM complex formation. We also identified that the peptide CD160 (25–44) also interacts with HVEM protein. This peptide contains the fragment CD160 (30–34), which was identified as a potential binding site of HVEM in HDX-MS experiment. These interactions seem to be important for the formation/stability of the CD160–HVEM complex structure. The Phe30^CD160^ and Val32^CD160^ amino acids are engaged in the interaction with key Tyr23^HVEM^ residue. Tyr23^HVEM^ residue was described as an important factor for the binding of BTLA [21] or gD [21, 25]. In our model of the CD160–HVEM structure, we could observe the interactions between Glu45^CD160^ and Tyr23^HVEM^. Unfortunately, the data obtained from ELISA and HDX-MS experiments do not indicate that Glu45^CD160^ is important for the complex stabilization.

The other two interacting fragments CD160 (61–80) and CD160 (73–92) are also very suitable for the HDX-MS experiment. The most important changes in deuteration level were observed for the amino acid sequences 70–75 and 76–87, which are the part of either CD160 (61–80) or CD160 (73–92), or both analyzed peptides. The presented model of HVEM–CD160 protein complex does not show the interactions between the fragment 70–75 of CD160 and HVEM protein, but suggest that the amino acids from 83 to 100 are important for the complex formation, in particular Arg89^CD160^ and Leu97^CD160^. Unfortunately, in our HDX-MS analysis (as a result of pepsin digestion) only one peptide (CD160 (88–101)) was detected and for which the small changes in deuteration level were observed. The affinity test showed that the CD160 (85–104) peptide interacts with HVEM protein, but results from ELISA test indicated that CD160 (85–104) interacts with plates in a nonspecific manner. For that reason the role of that fragment in CD160–HVEM interactions is not clear.

**Figure 8:**
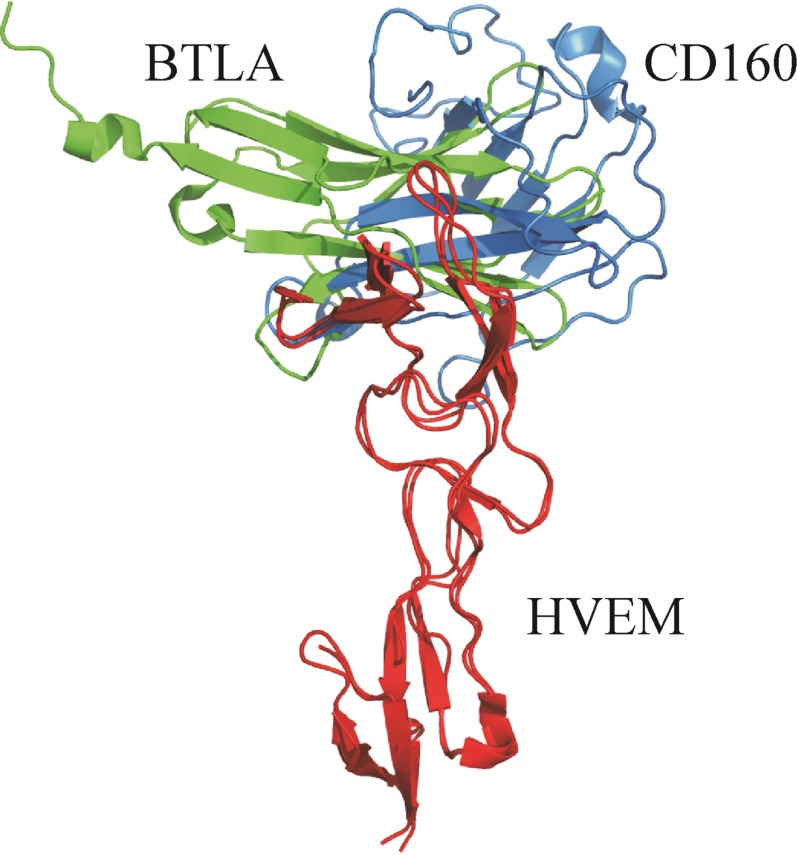
Comparison between the theoretical structure of the CD160 (blue)–HVEM (red) complex (UNRES model) with the crystal structure of BTLA (green)–HVEM (red) complex (PDB code:2AW2)

The HVEM engages in the BTLA–HVEM interaction with similar surfaces as in our predicted CD160–HVEM complex. In our model, the whole fragment 17–42^HVEM^ is involved in interactions with the CD160 protein. In the all-atom CD160–HVEM complex, after MD simulation, the fragment involved in the interaction is observed to be shorter: 17–39^HVEM^. This binding fragment in CD160–HVEM complex is almost identical to the most significant BTLA–HVEM binding fragment 14–39^HVEM^ [21]. The Tyr23^HVEM^ residue plays a crucial role in the BTLA–HVEM [21] interface and in the CD160–HVEM complex, according to our predictions. It indicates that the molecular recognition between HVEM (red) and BTLA (green) or HVEM and CD160 (blue) might be very similar (Figure 8). Although the binding site of the CD160 or BTLA proteins to the HVEM is very similar, in the 3D model (see Figure 8) it can be seen that the CD160 and BTLA proteins are displaced from each other as they are structurally different. The BTLA protein forms a very compact structure and more stable complex with the HVEM protein. This is confirmed by our nano differential scanning fluorimetry (nanoDSF) studies, through which we analyzed the thermal stability of both proteins (CD160 and BTLA) and both complexes (CD160–HVEM and BTLA–HVEM). The results obtained by us (unpublished data) indicate that the CD160 protein is less stable in comparison with BTLA and HVEM proteins. In addition, the CD160–HVEM complex is also found to be less stable than the BTLA–HVEM. The results of our experiments, in which we determined the binding sites between the CD160 and HVEM are in good agreement with the theoretical results, in which we obtained the structure of the CD160–HVEM complex without the experimental data. This high agreement between experimental and theoretical results indicate that the structure of the CD160–HVEM complex we proposed is robust. Our results can be useful in future to evaluate various possible strategies to block the HVEM protein in the CD160–HVEM or BTLA–HVEM interactions. However, additional studies are needed to better understand CD160–HVEM interactions.

## MATERIALS AND METHODS

### Sources of CD160 and HVEM proteins

The recombinant human CD160 protein was purchased from Novoprotein, USA (#CA35). Recombinant human protein HVEM with His Tag was purchased from Sino Biological Inc., China (#10334-H08H). Human HVEM-Fc protein was purchased from ACROBiosystems, USA (#HVM-H5258). For ELISA, CD160 protein was biotinylated using the EZ-link NHS-PEG4-Biotin (Thermo Fisher Scientific), according to the manufacturer's instruction. CD160 protein was deglycosylated using PNGase F enzyme (Promega) according to the manufacturer's instruction.

### Peptide synthesis and purification

The peptides were synthesized using standard protocols for the solid phase peptide synthesis [42]. The crude peptides were purified by RP-HPLC using a semipreparative Luna C8(2) column (20 × 250 mm, 5 μm) and 0.1% TFA in H_2_O (as solvent A) and 80% acetonitrile in water containing 0.08% TFA (as solvent B). For all peptides, a linear gradient from 20% B to 60% B in 140 mins was used; the flow rate was adjusted to 15 ml/min and the separation was monitored by UV absorbance at 222 nm. The purity of peptides was analyzed in linear gradient from 5 to 100% B in 60 min by using LCMS ESI/IT-TOF (Shimadzu).

### Preparation of microcolumn and affinity tests

The immobilization of HVEM protein in a microcolumn and affinity tests were performed as described previously [43].

### Enzyme-linked immunosorbent assay (ELISA)

ELISA tests were performed on a 96-well clear Nunc Immobilizer Streptavidin microplate (Thermo Scientific). In the first step, the plate was washed 5 times (200 μl/well) with PBS-T (5 mM Na_2_HPO_4_, 150 mM NaCl with addition of 0.3 M NaCl and 0.05% Tween-20, pH 7.4) and then coated with 100 μl of biotinylated peptides (1 μg/ml) in PBS-T for 1 hour at 25°C. Biotinylated CD160 protein (1 μg/ml) and PBS-T were used as positive and negative control, respectively. After washing with PBS-T, non-coated sites were blocked using the blocking solution (200 μl/well; Candor) for 2 h at 25°C. Then, after washing, 100 μl HVEM-Fc at different concentrations per well were added and incubated for 1 hour at 25°C. Goat antihuman IgG(H+L)–HRP (Bio-Rad) antibody (1:3000, 100 μl/well) was then added and incubated for 1 hour at 25°C. Finally, the binding of HVEM-Fc with peptides were determined by the addition of 100 μl/well of 3,3′,5,5′-tetramethylbenzidine (TMB, Thermo Scientific). Absorbance was measured using Infinite M200 Pro of Tecan at 650 nm and 492 nm. Statistical analysis of the results was performed using the one-way analysis of variance (GraphPad Prism 7).

### Hydrogen/deuterium exchange experiments

The method was performed as described before, with small modifications [44]. In the first step of the analysis, the list of CD160 peptides was created using a nondeuterated protein sample. Fifteen microliters portions of the CD160 protein stock solution (105 μM) were diluted by adding to 35 μl of PBS (5 mM Na_2_HPO_4_, 150 mM NaCl, pH 7.4). The sample was acidified by the addition of 10 μl H_2_O stop buffer (150 mM NaCl, 6 M guanidine hydrochloride, 1 M tris(2-carboxyethyl)phosphine (TCEP), 2 M glycine, pH 2.4). The sample was first digested offline for 1 min with 5 μl of protease type XIII from *Aspergillus saitoi* (48 μM, in 1% formic acid). Then, sample was digested online for 1.5 min using a 2.1 mm × 30 mm immobilized pepsin resin column (Poroszyme, ABI, Foster City, CA) with 0.07% formic acid in water as mobile phase (200 μl/min flow rate) kept at 13°C. Peptides were loaded onto the 2.1 mm × 5 mm C18 trapping column (ACQUITY BEH C18 VanGuard precolumn, 1.7 μm resin, Waters, Milford, MA). Trapped peptides were eluted onto a reversed phase C18 column (Acquity UPLC BEH, 1.0 × 100 mm, 1.7 μm resin, Waters, Milford, MA) using a 7–35% gradient of acetonitrile in 0.1% formic acid at 55 μl/min flow rate controlled by the nanoACQUITY binary solvent manager. Single run time was 13.5 min. Temperature of all fluidics, columns, and valves, with the exception of the pepsin digestion column kept at 13°C, was maintained at 0.5°C using the HDX Manager (Waters, Milford, MA). Outlet of the C18 column was directly coupled to the ion source of SYNAPT GS HDMS mass spectrometer (Waters, Milford, MA). Leucine-enkephalin (Sigma) was used for carrying out lock mass and activation. Mass spectra were acquired in MS^E^ mode over the *m*/*z* range of 50–2000. The spectrometer parameters were set as follows: ESI positive mode, capillary voltage 3 kV, sampling cone voltage 35 V, extraction cone voltage 3 V, source temperature 80°C, desolvation temperature 175°C, and desolvation gas flow 800 L/h. The spectrometer was calibrated using standard calibrating procedures. Peptides identification was performed with ProteinLynx Global Server software (PLGS, Waters, Milford, MA). We used a randomized database. The list of peptides, with peptide *m*/*z*, charge, retention time, and ion mobility/drift time was passed to the DynamX 3.0 HDX-MS data analysis software (Waters, Milford, MA).

Complex formation was initiated by mixing 84 μl of CD160 (c=105 μM) with 56 μl of protein HVEM (c=157 μM), 1:1 ratio in PBS. The mixture was incubated for 2 h at 25°C to enable the complex formation. Hydrogen/deuterium (H/D) exchange experiments were performed similarly as described for the nondeuterated sample. The reaction buffer was prepared using D_2_O (99.8% Cambridge Isotope Laboratories, Inc.), and pH was adjusted using DCl (Sigma) to 2.4 for stop buffer. 5 μl of protein stock was mixed with 45 μl D_2_O reaction buffer and the exchange reactions were performed for different time periods (10 s, 1 min, 5 min, 25 min, and 2 h) at room temperature. The exchange was quenched by reducing the pH to 2.4 by adding the reaction mixture to stop buffer cooled on ice (150 mM NaCl, 6 M guanidine hydrochloride, 1 M TCEP, 2 M glycine, pH 2.4). After quenching in the stop buffer, the sample was digested offline with *Aspergillus saitoi* protease XIII and the sample was manually injected into the nanoACQUITY (Waters, Milford, MA) UPLC system. Afterward, online pepsin digestion and liquid chromatography (LC) and MS analysis were conducted the same as described for nondeuterated samples.

Two control experiments were performed to exclude exchange artifacts. To assess the minimal level of exchange (in-exchange), 45 μl of D_2_O reaction buffer and 10 μl of stop buffer were mixed and cooled on ice, prior to the addition of 5 μl protein CD160 stock sample. Sample was immediately digested offline and online and subjected to LC/MS analysis, as the H/D exchange samples described above. Deuteration level measured for in-exchange control was calculated and denoted as 0% exchange $\left(M_{\text{ex}}^{0}\right)$. To assess the maximal level of exchange (out-exchange), the deuteration reaction was performed for 24 h, and subsequently quenched and processed as described above. Deuteration level measured for out-exchange control was calculated and denoted as 100% exchange $\left(M_{\text{ex}}^{100}\right)$. Each H/D exchange experiment was repeated four times, the presented results are the mean of these replicates.

### H/D exchange data analysis

The analysis was performed as described before [44]. The deuteration levels of each peptide were calculated automatically using DynamX 3.0 software. The peptide list was obtained from undeuterated samples using the PLGS program. The list was further filtered in the DynamX 3.0 program, using the following criteria: minimum intensity - 3000; minimum products per amino acid - 0,3; minimum score - 7,0; maximum MH+ error (ppm) - 10. All results from exchange and control experiments obtained from the automated analysis were subsequently verified manually. Overlapping or ambiguous isotopic envelopes were discarded.

The percentage of relative deuterium uptake (% Deuteration) of every peptide was calculated following the formula, where $\left(M_{\text{ex}}^{0}\right)$ and $\left(M_{\text{ex}}^{100}\right)$ indicate the minimum and maximum exchange values obtained in control experiments described before [44], respectively.

$$\%\text{Deuteration}=\frac{\left(M_{\text{ex}}-M_{\text{ex}}^{0}\right)}{\left(M_{\text{ex}}^{100}-M_{\text{ex}}^{0}\right)}\times 100$$

Final kinetic results, error bars, the values of the difference in exchange, and figures were calculated and plotted using the in-house scripts written in R language [45]. Error bars for fraction exchanged represent standard deviations calculated from at least three independent experiments. The difference in the Fraction Exchanged (Δ Fraction Exchanged) was calculated by subtracting the values for peptides in the complex from the values for the same peptides in the apo-state, with the error bars being calculated as the square root of the sum of the variances from compared states. *P*-values were calculated using Student's *t*-test for peptides after n time of HD exchange. Student's *t*-test for two independent samples with unequal variances and sample sizes (known as Welsh *t*-test) was carried out to evaluate differences between the same peptides in two different states, as described before [46].

### Model building of the CD160 protein

The structure of the CD160 protein was obtained initially by homology modeling and then was calculated in a coarse-grained force field. First, structural models were obtained with the use of I-TASSER server [47–50]. I-TASSER homology modeling was based on templates of the highest significance in the threading alignments, and the ten best templates were used to build the CD160 models. There were no homologous template structures for the *C*-terminal part of the protein (119–159 fragments). This part of the structure was therefore obtained in the next step by using the multiplexed replica exchange molecular dynamics (see next indention). For each target, I-TASSER simulations generate a large ensemble of conformation, which were divided into clusters based on their structural similarity. The final five structures were average structures corresponding to the five largest structure clusters. For those structures, a C-score, the confidence score for estimating the quality of predicted models by I-TASSER, were calculated. The C-score is typically in the range of −5 and 2 [50, 51], where a higher C-score value a higher confidence in a model. The CD160 model with the highest C-score was selected for the structure calculation in a coarse-grained force field.

To get folded structures of the full length CD160 protein, the I-TASSER model in the multiplexed replica exchange molecular dynamics trajectories (MREMD) [51, 52] with the coarse-grained UNRES (from UNited RESidue) [53, 54] force field were calculated. The simulations were performed with the use of the dynamic fragment assembly (DFA) technique, in which knowledge-based information corresponding to secondary structure, inter-residue-contacts, and local structure is incorporated into a target function as additional energy terms [55, 56]. Subsequently, the weighted-histogram analysis method (WHAM) was applied to calculate the relative free energy of each structure of the last part of the MREMD simulation [57, 58] and then cluster analysis was used to obtain clusters with the lowest free energies. Finally, the average structure from each cluster was converted to an all-atom model and refined by using restrained molecular dynamics simulations with the AMBER14 all-atom force field (3.7 ns). Final conformations were picked based on the following criteria:

- protons in the CD160 protein, exchanged on deuterium in HDX-MS experiment are situated in the unstructured fragments of the protein and in the solvent-exposed part of the protein. This was the key information during the selection of the theoretically appointed structure of the CD160 protein,
- the fragments binding to HVEM, indicated by HDX-MS technique, should be in close contact and should be located in the solvent-exposed areas,
- correct positions of disulfide bonds in CD160 protein model,
- proper topology of CD160 model in a IgV immunoglobulin fold.

The best 3D model of CD160 protein among all calculated structures was validated using the following programs PROCHECK [59], VERIFY3D [60], and ProSA-Web (Protein Structure Analysis) [61]. For the analysis details see description in Supplementary Materials (see Supplementary Figure 6).

### CD160–HVEM complex formation by molecular docking

The best 3D model of CD160 protein was used for protein docking with HVEM protein by using coarse-grained UNRES force field [62, 63] with newly developed restrains on tertiary structures of monomers [64]. The experimental data was not used during the docking procedure. HVEM coordinates were used from the structure of the BTLA-HVEM complex (PDB code: 2AW2). As a starting structure a randomly oriented proteins were used and multiplex replica exchange molecular dynamics trajectories were calculated (MREMD) [52]. After simulation, the weighted histogram analysis and clustering of the obtained structures was performed. For the dominant cluster (with lowest free energy) contact map analysis was performed [65]. In the calculations the cuttoff1 was set to 8 and the cutoff2 was set to 10. The most probable cluster was converted to all-atom structure with use of PULCHRA [66] software. Afterward, to refine structures which were derived from coarse-grained models and check the stability of the complex, the all-atom molecular dynamics (MD) simulation in AMBER ff14SB force field was performed [67]. Simulations were performed in explicit solvent (TIP3PBOX water model) in water box and in standard temperature (298K). Size of the water box was set as 15Å of water molecules in each side of the protein. Interactions in the CD160–HVEM protein were analyzed by using YASARA program [68].

## SUPPLEMENTARY MATERIALS FIGURES AND TABLES



## Abbreviations

Abu: 2-aminobutyric acid
BTLA: B- and T-lymphocyte attenuator
CD160: cluster of differentiation 160
CRD: cysteine-rich domain
CTLA-4: cytotoxic T-lymphocyte-associated protein 4
ELISA: enzyme-linked immunosorbent assay
gD: glycoprotein D
GPI: glycosylphosphatidylinositol
HDX-MS: hydrogen/deuterium exchange combined to mass spectrometry
HRP: horseradish peroxidase
HVEM: herpes virus entry mediator
HSV: herpes simplex virus
IgSF: immunoglobulin-like superfamily
LTα: lymphotoxin α
LIGHT: homologous to lymphotoxins, exhibits inducible expression, and competes with HSV glycoprotein D for herpes virus entry mediator, a receptor expressed by T lymphocytes
MHC: major histocompatibility complex
NK: natural killer
PBS: phosphate-buffered saline
PD-1: programmed cell death 1
IgV: immunoglobulin-like V-type
TNFRSF: tumor necrosis factor receptor superfamily
TNFSF: tumor necrosis factor superfamily

## References

[1] Kim ES, Kim JE, Patel MA, Mangraviti A, Ruzevick J, Lim M (2016). Immune Checkpoint Modulators: An Emerging Antiglioma Armamentarium. J Immunol Res.

[2] Cai G, Freeman GJ (2009). The CD160, BTLA, LIGHT/HVEM pathway: a bidirectional switch regulating T-cell activation. Immunol Rev.

[3] Pico de Coaña Y, Choudhury A, Kiessling R (2015). Checkpoint blockade for cancer therapy: revitalizing a suppressed immune system. Trends Mol Med.

[4] Brunet JF, Denizot F, Luciani MF, Roux-Dosseto M, Suzan M, Mattei MG, Golstein P (1987). A new member of the immunoglobulin superfamily—CTLA-4. Nature.

[5] Linsley PS, Brady W, Urnes M, Grosmaire LS, Damle NK, Ledbetter JA (1991). CTLA-4 is a second receptor for the B cell activation antigen B7. J Exp Med.

[6] Freeman GJ, Long AJ, Iwai Y, Bourque K, Chernova T, Nishimura H, Fitz LJ, Malenkovich N, Okazaki T, Byrne MC, Horton HF, Fouser L, Carter L (2000). Engagement of the PD-1 immunoinhibitory receptor by a novel B7 family member leads to negative regulation of lymphocyte activation. J Exp Med.

[7] Latchman Y, Wood CR, Chernova T, Chaudhary D, Borde M, Chernova I, Iwai Y, Long AJ, Brown JA, Nunes R, Greenfield EA, Bourque K, Boussiotis VA (2001). PD-L2 is a second ligand for PD-1 and inhibits T cell activation. Nat Immunol.

[8] Mahnke K, Enk AH (2016). TIGIT-CD155 Interactions in Melanoma: A Novel Co-Inhibitory Pathway with Potential for Clinical Intervention. J Invest Dermatol.

[9] Webb GJ, Hirschfield GM, Lane PJ (2016). OX40, OX40L and Autoimmunity: a Comprehensive Review. Clin Rev Allergy Immunol.

[10] Ju Y, Hou N, Meng J, Wang X, Zhang X, Zhao D, Liu Y, Zhu F, Zhang L, Sun W, Liang X, Gao L, Ma C (2010). T cell immunoglobulin- and mucin-domain-containing molecule-3 (Tim-3) mediates natural killer cell suppression in chronic hepatitis B. J Hepatol.

[11] Croft M (2005). The evolving crosstalk between co-stimulatory and co-inhibitory receptors: HVEM-BTLA. Trends Immunol.

[12] Sedy JR, Gavrieli M, Potter KG, Hurchla MA, Lindsley RC, Hildner K, Scheu S, Pfeffer K, Ware CF, Murphy TL, Murphy KM (2005). B and T lymphocyte attenuator regulates T cell activation through interaction with herpesvirus entry mediator. Nat Immunol.

[13] Cai G, Anumanthan A, Brown JA, Greenfield EA, Zhu B, Freeman GJ (2008). CD160 inhibits activation of human CD4+ T cells through interaction with herpesvirus entry mediator. Nat Immunol.

[14] Mauri DN, Ebner R, Montgomery RI, Kochel KD, Cheung TC, Yu GL, Ruben S, Murphy M, Eisenberg RJ, Cohen GH, Spear PG, Ware CF (1998). LIGHT, a new member of the TNF superfamily, and lymphotoxin α are ligands for herpesvirus entry mediator. Immunity.

[15] Ware CF (2008). Targeting lymphocyte activation through the lymphotoxin and LIGHT pathways. Immunol Rev.

[16] Cheung TC, Steinberg MW, Oborne LM, Macauley MG, Fukuyama S, Sanjo H, D'Souza C, Norris PS, Pfeffer K, Murphy KM, Kronenberg M, Spear PG, Ware CF (2009). Unconventional ligand activation of herpesvirus entry mediator signals cell survival. Proc Natl Acad Sci U S A.

[17] Steinberg MW, Cheung TC, Ware CF (2011). The signaling networks of the herpesvirus entry mediator (TNFRSF14) in immune regulation. Immunol Rev.

[18] Kwon BS, Tan KB, Ni J, Oh KO, Lee ZH, Kim KK, Kim YJ, Wang S, Gentz R, Yu GL, Harrop J, Lyn SD, Silverman C (1997). A newly identified member of the tumor necrosis factor receptor superfamily with a wide tissue distribution and involvement in lymphocyte activation. J Biol Chem.

[19] Hsu H, Solovyev I, Colombero A, Elliott R, Kelley M, Boyle WJ (1997). ATAR, a novel tumor necrosis factor receptor family member, signals through TRAF2 and TRAF5. J Biol Chem.

[20] Bodmer JL, Schneider P, Tschopp J (2002). The molecular architecture of the TNF superfamily. Trends Biochem Sci.

[21] Compaan DM, Gonzalez LC, Tom I, Loyet KM, Eaton D, Hymowitz SG (2005). Attenuating lymphocyte activity: the crystal structure of the BTLA-HVEM complex. J Biol Chem.

[22] Gonzalez LC, Loyet KM, Calemine-Fenaux J, Chauhan V, Wranik B, Ouyang W, Eaton DL (2005). A coreceptor interaction between the CD28 and TNF receptor family members B and T lymphocyte attenuator and herpesvirus entry mediator. Proc Natl Acad Sci U S A.

[23] Whitbeck JC, Peng C, Lou H, Xu R, Willis SH, Ponce de Leon M, Peng T, Nicola AV, Montgomery RI, Warner MS, Soulika AM, Spruce LA, Moore WT (1997). Glycoprotein D of herpes simplex virus (HSV) binds directly to HVEM, a member of the tumor necrosis factor receptor superfamily and a mediator of HSV entry. J Virol.

[24] Connolly SA, Landsburg DJ, Carfi A, Wiley DC, Eisenberg RJ, Cohen GH (2002). Structure-based analysis of the herpes simplex virus glycoprotein D binding site present on herpesvirus entry mediator HveA (HVEM). J Virol.

[25] Carfí A, Willis SH, Whitbeck JC, Krummenacher C, Cohen GH, Eisenberg RJ, Wiley DC (2001). Herpes simplex virus glycoprotein D bound to the human receptor HveA. Mol Cell.

[26] Connolly SA, Landsburg DJ, Carfi A, Wiley DC, Cohen GH, Eisenberg RJ (2003). Structure-based mutagenesis of herpes simplex virus glycoprotein D defines three critical regions at the gD-HveA/HVEM binding interface. J Virol.

[27] Kojima R, Kajikawa M, Shiroishi M, Kuroki K, Maenaka K (2011). Molecular basis for herpesvirus entry mediator recognition by the human immune inhibitory receptor CD160 and its relationship to the cosignaling molecules BTLA and LIGHT. J Mol Biol.

[28] Giustiniani J, Bensussan A, Marie-Cardine A (2009). Identification and characterization of a transmembrane isoform of CD160 (CD160-TM), a unique activating receptor selectively expressed upon human NK cell activation. J Immunol.

[29] El-Far M, Pellerin C, Pilote L, Fortin JF, Lessard IA, Peretz Y, Wardrop E, Salois P, Bethell RC, Cordingley MG, Kukolj G (2014). CD160 isoforms and regulation of CD4 and CD8 T-cell responses. J Transl Med.

[30] Barakonyi A, Rabot M, Marie-Cardine A, Aguerre-Girr M, Polgar B, Schiavon V, Bensussan A, Le Bouteiller P (2004). Cutting edge: engagement of CD160 by its HLA-C physiological ligand triggers a unique cytokine profile secretion in the cytotoxic peripheral blood NK cell subset. J Immunol.

[31] Rudd PM, Joao HC, Coghill E, Fiten P, Saunders MR, Opdenakker G, Dwek RA (1994). Glycoforms modify the dynamic stability and functional activity of an enzyme. Biochemistry.

[32] De Rosa MC, Pirolli D, Bozzi M, Sciandra F, Giardina B, Brancaccio A (2011). A second Ig-like domain identified in dystroglycan by molecular modelling and dynamics. J Mol Graph Model.

[33] Wiederstein M, Sippl MJ (2007). ProSA-web: interactive web service for the recognition of errors in three-dimensional structures of proteins. Nucleic Acids Res.

[34] Bork P, Holm L, Sander C (1994). The immunoglobulin fold. Structural classification, sequence patterns and common core. J Mol Biol.

[35] Peretz Y, He Z, Shi Y, Yassine-Diab B, Goulet JP, Bordi R, Filali-Mouhim A, Loubert JB, El-Far M, Dupuy FP, Boulassel MR, Tremblay C, Routy JP (2012). CD160 and PD-1 co-expression on HIV-specific CD8 T cells defines a subset with advanced dysfunction. PLoS Pathog.

[36] Viganò S, Banga R, Bellanger F, Pellaton C, Farina A, Comte D, Harari A, Perreau M (2014). CD160-associated CD8 T-cell functional impairment is independent of PD-1 expression. PLoS Pathog.

[37] Sedy JR, Veny M, Nguyen J, Balmert MO, Niemela N, Norris PS, Ware CF (2016). Targeting the HVEM-BTLA-CD160-LIGHT network in Psoriasis. J Immunol.

[38] Chabot S, Jabrane-Ferrat N, Bigot K, Tabiasco J, Provost A, Golzio M, Noman MZ, Giustiniani J, Bellard E, Brayer S, Aguerre-Girr M, Meggetto F, Giuriato S (2011). A novel antiangiogenic and vascular normalization therapy targeted against human CD160 receptor. J Exp Med.

[39] Konermann L (2016). Heavy lessons in protein allostery. Nat Struct Mol Biol.

[40] Prądzińska M, Behrendt I, Astorga-Wells J, Manoilov A, Zubarev RA, Kołodziejczyk AS, Rodziewicz-Motowidło S, Czaplewska P (2016). Application of amide hydrogen/deuterium exchange mass spectrometry for epitope mapping in human cystatin C. Amino Acids.

[41] Butler JE (2000). Enzyme-linked immunosorbent assay. J Immunoassay.

[42] Fields GB, Noble RL (1990). Solid phase peptide synthesis utilizing 9-fluorenylmethoxycarbonyl amino acids. Int J Pept Protein Res.

[43] Spodzieja M, Lach S, Iwaszkiewicz J, Cesson V, Kalejta K, Olive D, Michielin O, Speiser DE, Zoete V, Derré L, Rodziewicz-Motowidło S (2017). Design of short peptides to block BTLA/HVEM interactions for promoting anticancer T-cell responses. PLoS One.

[44] Premchandar A, Mücke N, Poznański J, Wedig T, Kaus-Drobek M, Herrmann H, Dadlez M (2016). Structural dynamics of the vimentin coiled-coil contact regions involved in filament assembly as revealed by hydrogen-deuterium exchange. J Biol Chem.

[45] The R Project for Statistical Computing. https://www.r-project.org/.

[46] Skrajna A, Yang XC, Tarnowski K, Fituch K, Marzluff WF, Dominski Z, Dadlez M (2016). Mapping the Interaction Network of Key Proteins Involved in Histone mRNA Generation: A Hydrogen/Deuterium Exchange Study. J Mol Biol.

[47] Zhang Y (2008). I-TASSER server for protein 3D structure prediction. BMC Bioinformatics.

[48] Roy A, Kucukural A, Zhang Y (2010). I-TASSER: a unified platform for automated protein structure and function prediction. Nat Protoc.

[49] Yang J, Zhang Y (2015). I-TASSER server: new development for protein structure and function predictions. Nucleic Acids Res.

[50] Yang J, Yan R, Roy A, Xu D, Poisson J, Zhang Y (2015). The I-TASSER Suite: protein structure and function prediction. Nat Methods.

[51] Khalili M, Liwo A, Rakowski F, Grochowski P, Scheraga HA (2005). Molecular dynamics with the united-residue model of polypeptide chains. I. Lagrange equations of motion and tests of numerical stability in the microcanonical mode. J Phys Chem B.

[52] Czaplewski C, Kalinowski S, Liwo A, Scheraga HA (2009). Application of Multiplexed Replica Exchange Molecular Dynamics to the UNRES Force Field: tests with α and α+β Proteins. J Chem Theory Comput.

[53] Rojas A, Czaplewski C, Liwo A, Makowski M, Ołdziej S, Kazmierkiewicz R, Scheraga H, Murarka R (2008). Simulation of Protein Structure and Dynamics with the Coarse-Grained UNRES Force Field. Coarse-Graining of Condensed Phase and Biomolecular Systems.

[54] Liwo A, Khalili M, Czaplewski C, Kalinowski S, Ołdziej S, Wachucik K, Scheraga HA (2007). Modification and optimization of the united-residue (UNRES) potential energy function for canonical simulations. I. Temperature dependence of the effective energy function and tests of the optimization method with single training proteins. J Phys Chem B.

[55] Lee J, Park K, Lee J (2002). Full optimization of linear parameters of a united residue protein potential. J Phys Chem B.

[56] Sasaki TN, Cetin H, Sasai M (2008). A coarse-grained Langevin molecular dynamics approach to de novo protein structure prediction. Biochem Biophys Res Commun.

[57] Kumar S, Rosenberg JM, Bouzida D, Swendsen RH, Kollman PA (1992). The weighted histogram analysis method for free-energy calculations on biomolecules. I. The method. J Comput Chem.

[58] Ołdziej S, Liwo A, Czaplewski C, Pillardy J, Scheraga HA (2004). Optimization of the UNRES Force Field by Hierarchical Design of the Potential-Energy Landscape. 2. Off-Lattice Tests of the Method with Single Proteins. J Phys Chem B.

[59] Laskowski RA, MacArthur MW, Moss DS, Thornton JM (1993). PROCHECK: a program to check the stereochemical quality of protein structures. J Appl Crystallogr.

[60] Eisenberg D, Lüthy R, Bowie JU (1997). VERIFY3D: assessment of protein models with three-dimensional profiles. Methods Enzymol.

[61] ProSA-web - Protein Structure Analysis. https://prosa.services.came.sbg.ac.at/prosa.php.

[62] Krupa P, Hałabis A, Żmudzińska W, Ołdziej S, Scheraga HA, Liwo A (2017). Maximum Likelihood Calibration of the UNRES Force Field for Simulation of Protein Structure and Dynamics. J Chem Inf Model.

[63] Krupa P, Sieradzan AK, Rackovsky S, Baranowski M, Ołldziej S, Scheraga HA, Liwo A, Czaplewski C (2013). Improvement of the treatment of loop structures in the UNRES force field by inclusion of coupling between backbone- and side-chain-local conformational states. J Chem Theory Comput.

[64] Krupa P, Mozolewska MA, Joo K, Lee J, Czaplewski C, Liwo A (2015). Prediction of Protein Structure by Template-Based Modeling Combined with the UNRES Force Field. J Chem Inf Model.

[65] Peng X, Sieradzan AK, Niemi AJ (2016). Thermal unfolding of myoglobin in the Landau-Ginzburg-Wilson approach. Phys Rev E.

[66] Rotkiewicz P, Skolnick J (2008). Fast procedure for reconstruction of full-atom protein models from reduced representations. J Comput Chem.

[67] Maier JA, Martinez C, Kasavajhala K, Wickstrom L, Hauser KE, Simmerling C (2015). ff14SB: Improving the Accuracy of Protein Side Chain and Backbone Parameters from ff99SB. J Chem Theory Comput.

[68] Krieger E, Koraimann G, Vriend G (2002). Increasing the precision of comparative models with YASARA NOVA—a self-parameterizing force field. Proteins.

