# Supplementary material for: A structural model of the immune checkpoint CD160–HVEM complex derived from HDX-mass spectrometry and molecular modeling
Source: Oncotarget. 2019 Jan 11;10(4):536–50. doi: 10.18632/oncotarget.26570 (PMC6355189; doi:10.18632/oncotarget.26570)
Supplement: Supplementary file 1 [file oncotarget-10-536-s001.pdf]

A structural model of the immune checkpoint CD160–HVEM complex derived from HDX-mass spectrometry and molecular modeling

SUPPLEMENTARY MATERIALS

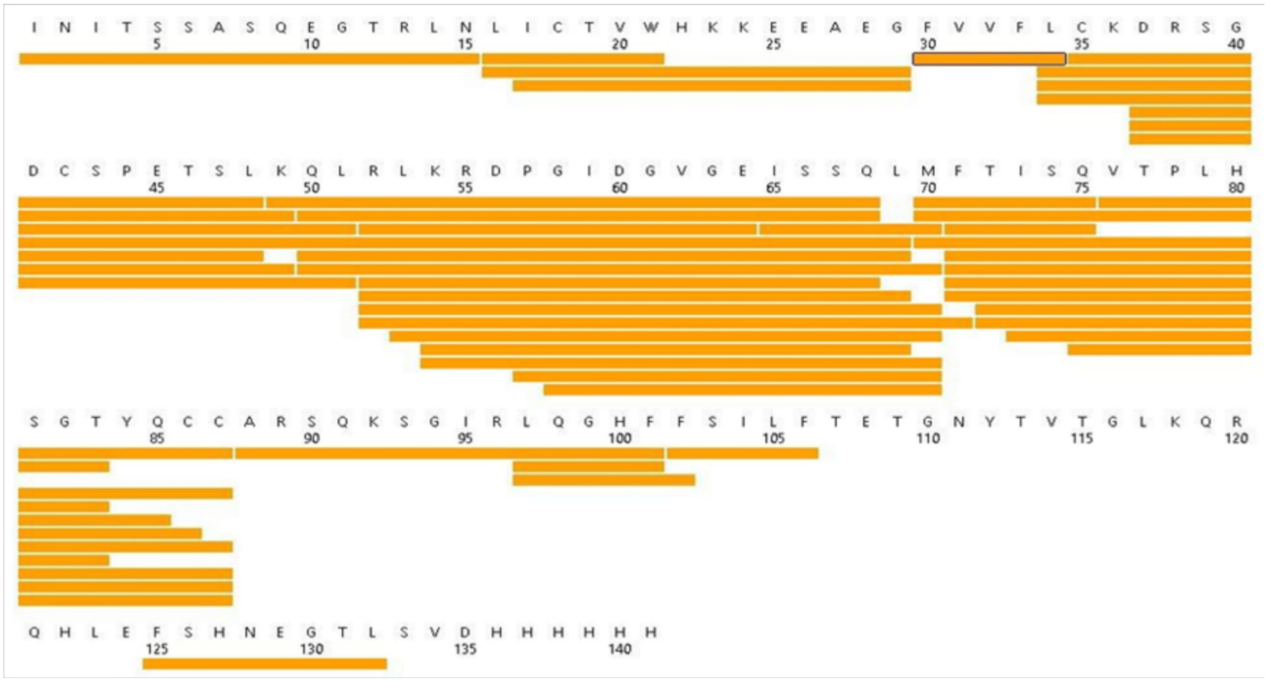

Supplementary Figure 1: Peptides detected by LC-MS after pepsin digestion of the CD160 protein. CD160 containing two additional amino acids in C-terminal and a His tag were used.

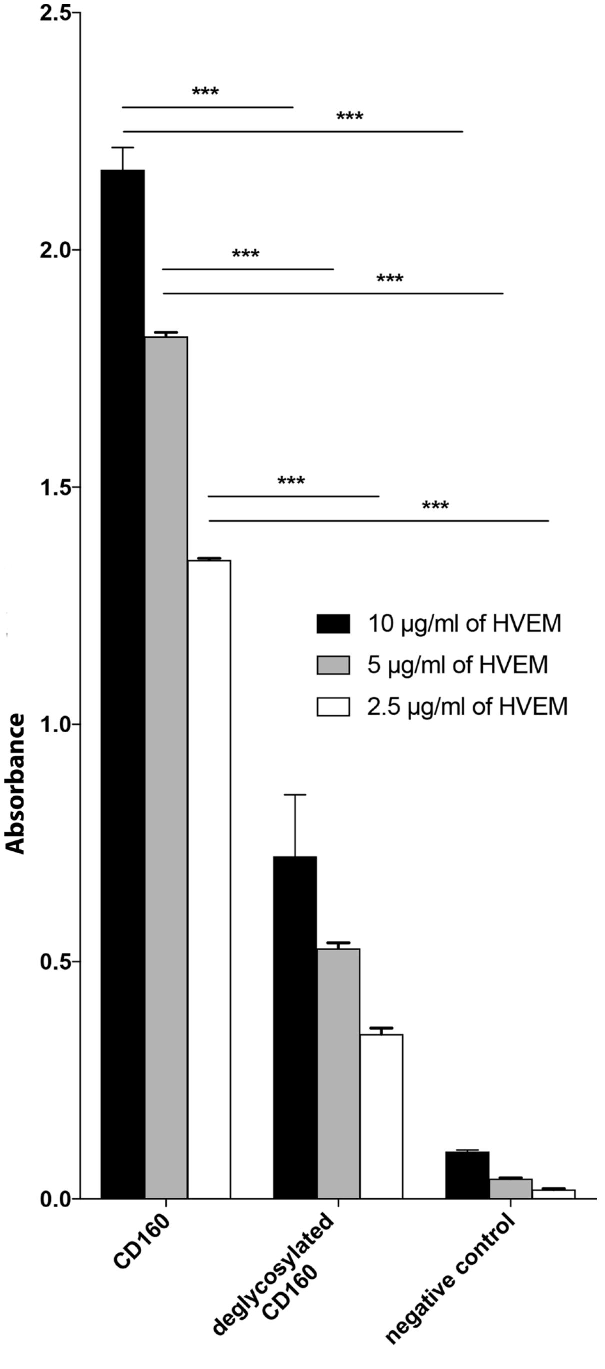

Supplementary Figure 2: ELISA test for native and deglycosylated CD160 protein. In assay native and deglycosylated CD160 protein (concentration 5 µg/ml) were immobilized in the nickel coated plate. Then, after blocking with 5% BSA, the plate was incubated with HVEM-Fc (concentration 10 µg/ml). Next, goat anti-human IgG-HRP conjugate (Bio Rad) was added. Then, the plate was incubated with TMB and the absorbance was read at 650 nm using a microplate reader (Infinite Pro Tecan).

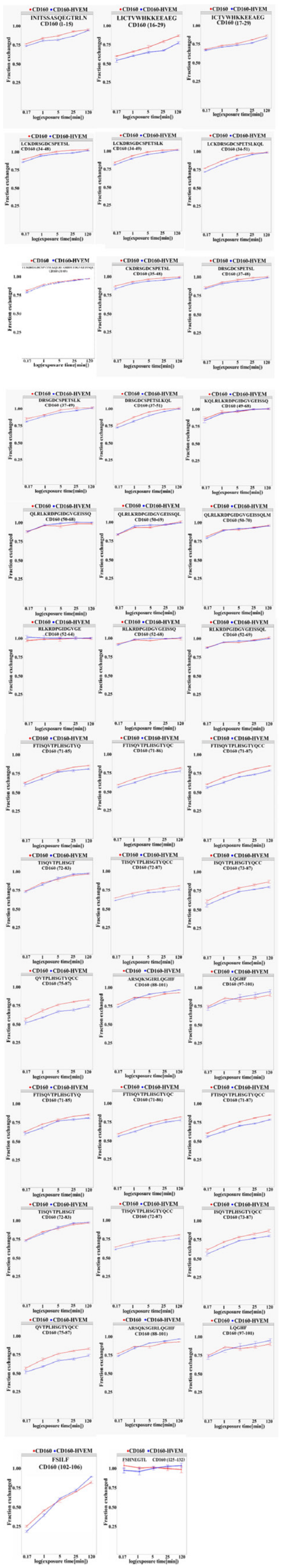

Supplementary Figure 3: Regions of CD160 protein without changes in deuteration level upon interaction with HVEM protein. CD160 in complex with HVEM (blue line) and free CD160 protein (red line). Deuteration was measured after incubation in deuterated PBS for 10 s, 1 min, 5 min, 25 min, 2 h and is shown on a logarithmic scale.

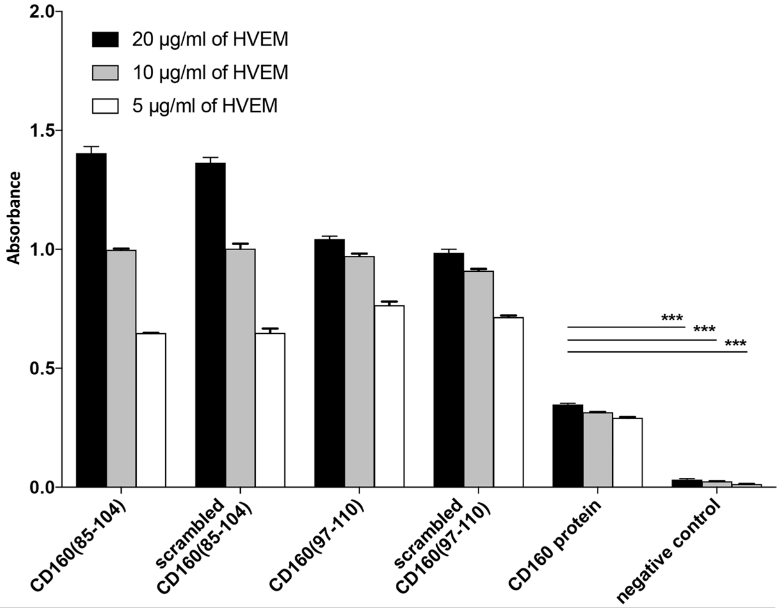

Peptide	Amino acid sequence
**Scrambled CD160 (85-104)**	Ac-ISICSQRRGQAHSQLGFCKF-NH_2
**Scrambled CD160 (97-110)**	Ac-GTSILQETFHGFFL-NH_2

Supplementary Figure 4: ELISA test results for binding of CD160 (85-104), CD160 (97-110), scrambled peptides to HVEM-Fc protein and amino acids sequence of scrambled peptides.

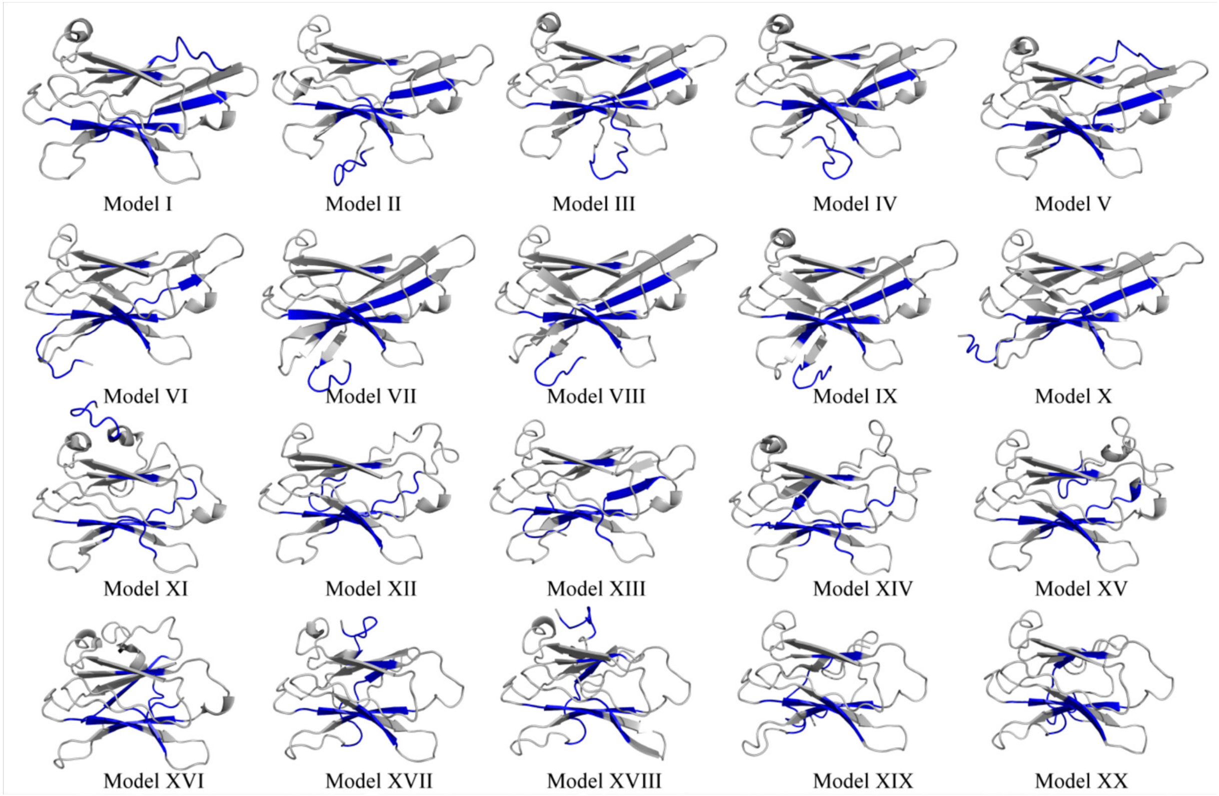

Supplementary Figure 5: 3D-structures of CD160 protein. Structural models generated by UNRES program. In blue color are signed the residues which not exchange their protons in HDX-MS experiment.

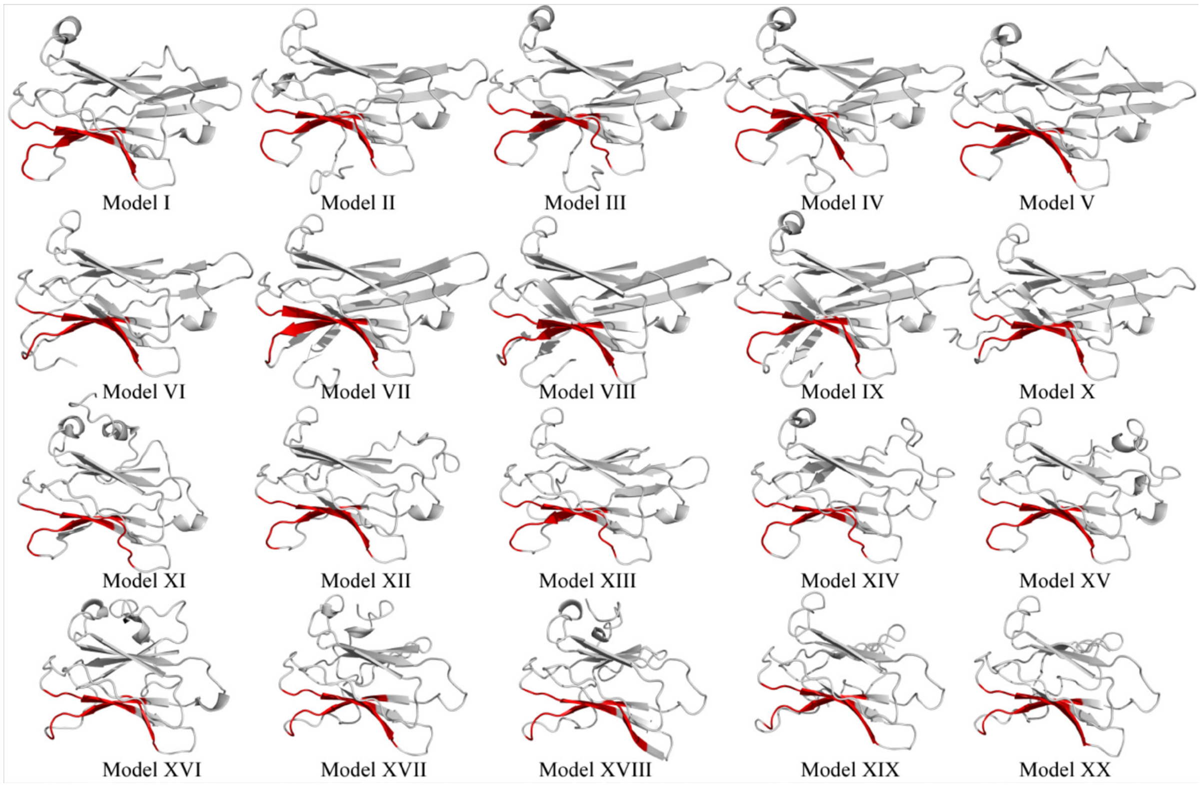

Supplementary Figure 6: 3D-structures of CD160 protein. Structural models generated by UNRES program. In red color are signed the residues which are epitopes in CD160 protein.

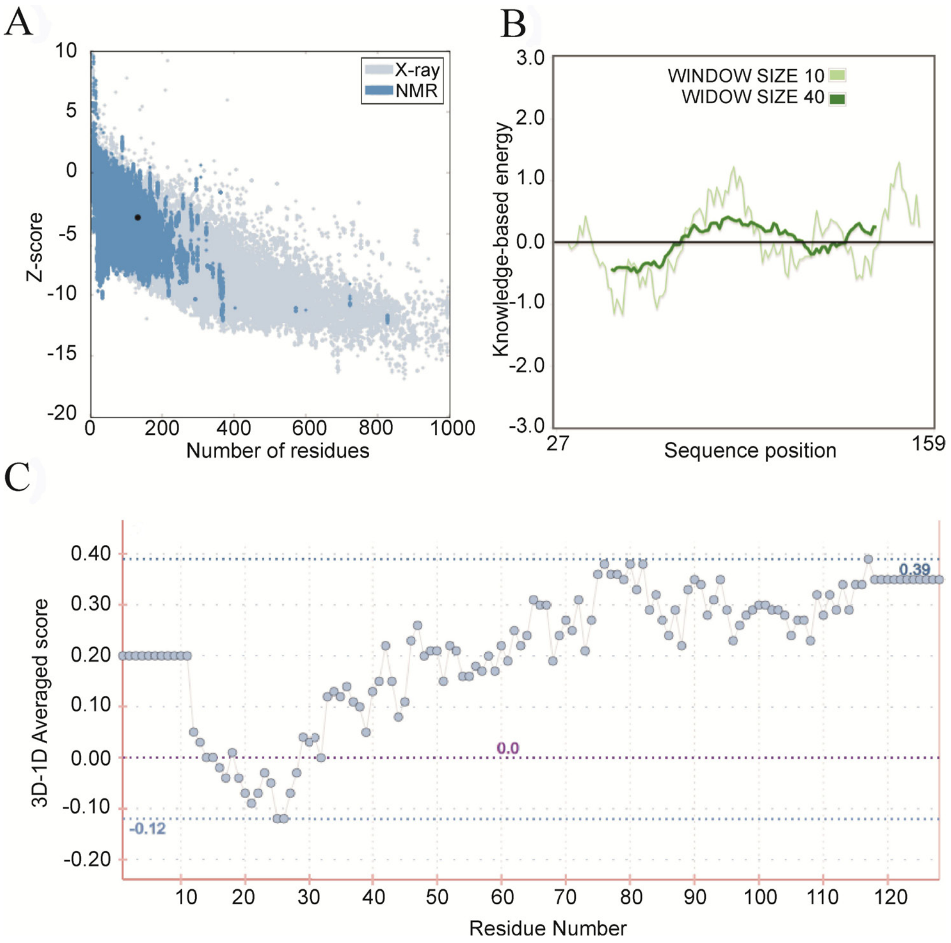

Supplementary Figure 7: Quality assessment of the modeled CD160 protein (Model XX). (**A**) ProSA-web z-score plot. Z-score value for CD160 protein (Supplementary Figure 6A) is displayed in a plot that contains the z-scores of all experimentally determined protein chains in current PDB. In this plot, groups of structures from different sources (X-ray, NMR) are distinguished by different colors. It can be used to check whether the z-score of the input structure is within the range of scores typically found for native proteins of similar size. (**B**) Energy plot from ProSA-web of the UNRES model. Plot of energy is a running average of knowledge based potential energy. plot shows local model quality by plotting energies as a function of amino acid sequence position I for CD160 protein. In general, positive values correspond to problematic or erroneous parts of the input structure. A plot of single residue energies usually contains large fluctuations and is of limited value for model evaluation. Hence the plot is smoothed by calculating the average energy over each 40-residue fragment s(i,i+39), which is then assigned to the 'central' residue of the fragment at position i+19 (Supplementary Figure 6B, thick line). A second line with a smaller window size of 10 residues is shown in the background of the plot (Supplementary Figure 6B, thin line). (**C**) Local model quality VERIFY-3D plots of UNRES model as a function of residue index. In Verify 3D Results plot (Supplementary Figure 6C) 67.97% of the residues had an averaged 3D-1D score >= 0.2 and less than 65% of the amino acids have scored >= 0.2 in the 3D/1D profile.

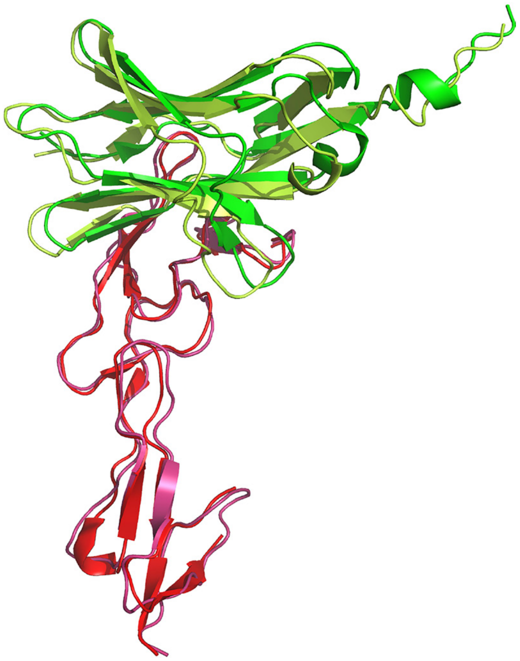

Supplementary Figure 8: Validation of UNRES docking method with use of BTLA-HVEM complex, using random orientation of both proteins with respect to each other. UNRES docking was able to find correct protein orientation with average rmsd of third cluster of 1.68A.

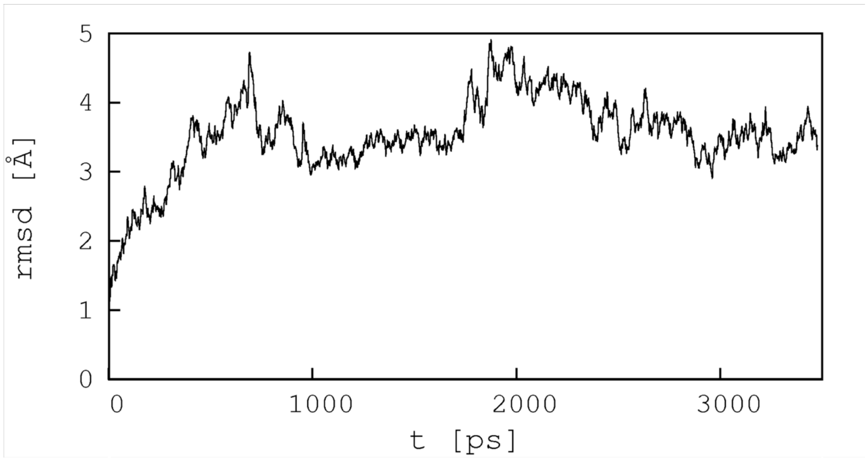

Supplementary Figure 9: Root-mean-square deviation as a function of time in all-atom simulations of HVEM-CD160 complex.

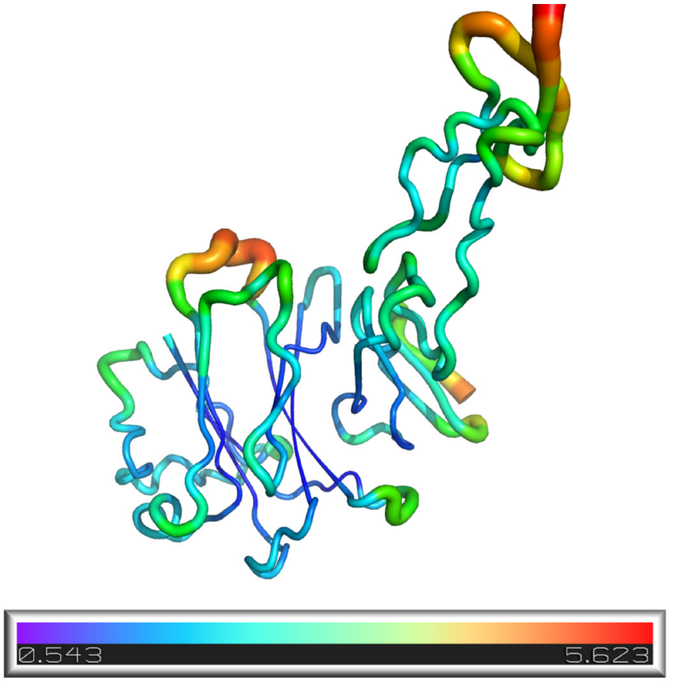

Supplementary Figure 10: Fluctuations of CD160-HVEM complex during MD calculations.

Supplementary Table 1: *P*-values calculated using Student's *t*-test for peptides after *n* time of HD exchange

Amino acid sequence	Amino acid position in protein	*p*_value				
		10 s	1 min	5 min	25 min	2 h
INITSSASQEGTRLN	1–15	0.0006	0.0150	0.0006	0.0002	0.2757
LICTVW	16–21	0.0011	0.0006	0.0000	0.0001	NA
LICTVWHKKEEAEG	16–29	0.0143	0.0007	0.0040	0.0000	0.0001
ICTVWHKKEEAEG	17–29	0.0140	0.0024	0.0245	0.0003	0.0016
FVVFL	30–34	0.0027	0.0019	0.0003	0.0005	0.0000
LCKDRSGDCSPETSL	34–48	0.0001	0.0204	0.0081	0.0031	0.1469
LCKDRSGDCSPETSLK	34–49	0.0032	0.0013	0.0022	0.0150	0.2914
LCKDRSGDCSPETSLKQL	34–51	0.0001	0.0002	0.0007	0.0104	0.9872
LCKDRSGDCSPETSLKQLRLKRDP GIDGVGEISSQL	34–69	0.0127	0.0544	0.0215	0.0199	0.9872
CKDRSGDCSPETSL	35–48	0.0095	0.0083	0.0067	0.0111	0.0379
DRSGDCSPETSL	37–48	0.0905	0.1626	0.0216	0.0043	0.1290
DRSGDCSPETSLK	37–49	0.0112	0.0655	0.0099	0.0102	0.7513
DRSGDCSPETSLKQL	37–51	0.0179	0.0006	0.0037	0.0014	0.2694
KQLRLKRDPGIDGVGEISSQ	49–68	0.2136	0.1643	0.2947	0.9253	0.7953
QLRLKRDPGIDGVGEISSQ	50–68	0.7800	0.4068	0.0362	0.0346	0.0515
QLRLKRDPGIDGVGEISSQL	50–69	0.6997	0.0880	0.0074	0.9391	0.0890
QLRLKRDPGIDGVGEISSQLM	50–70	0.0012	0.1465	0.0200	0.1026	0.4115
RLKRDPGIDGVGE	52–64	0.0151	0.3484	0.3989	0.9999	0.4716
RLKRDPGIDGVGEISSQ	52–68	0.0151	0.2236	0.0017	0.5441	0.3761
RLKRDPGIDGVGEISSQL	52–69	0.1558	0.6932	0.0048	0.0318	0.0879
RLKRDPGIDGVGEISSQLM	52–70	0.5629	0.8272	0.0075	0.0662	0.0944
LKRDPGIDGVGEISSQLM	53–70	0.2343	0.8429	0.1944	0.2253	0.1370
KRDPGIDGVGEISSQL	54–69	0.1116	0.4924	0.0341	0.0011	0.3856
KRDPGIDGVGEISSQLM	54–70	0.7744	0.1589	0.0135	0.4281	0.1076
PGIDGVGEISSQLM	57–70	0.1308	0.7798	0.3249	0.1926	0.0120
MFTISQVTPLHSGT	70–83	0.0672	0.1845	0.0788	0.2655	0.0039
MFTISQVTPLHSGTYQCC	70–87	0.0177	0.0111	0.0158	0.0009	0.0000
FTISQ	71–75	0.8244	0.4032	0.9148	0.0009	0.0000
FTISQVTPLHSGT	71–83	0.0168	0.0091	0.8884	0.2007	0.0100
FTISQVTPLHSGTYQ	71–85	0.0599	0.0064	0.1604	0.0082	0.0004
FTISQVTPLHSGTYQC	71–86	0.0013	0.0007	0.0014	0.0054	0.0184
FTISQVTPLHSGTYQCC	71–87	0.0027	0.0008	0.0001	0.0006	0.0001
TISQVTPLHSGT	72–83	0.3446	0.0191	0.3034	0.0663	0.1577
TISQVTPLHSGTYQCC	72–87	0.0002	0.0286	0.0010	0.0007	0.0028
ISQVTPLHSGTYQCC	73–87	0.0143	0.0002	0.0008	0.0026	0.0068
QVTPLHSGTYQCC	75–87	0.0107	0.0008	0.0002	0.0001	0.0023
VTPLHSGTYQCC	76–87	0.0027	0.0004	0.0001	0.0028	0.0000
ARSQKSGIRLQGHF	88–101	0.0017	0.0548	0.0555	0.0002	0.0000
LQGHF	97–101	0.3232	0.0548	0.2586	0.0439	0.0205
FSILF	102–106	0.0003	0.0023	0.3057	0.0649	0.0017
FSHNEGTL	125–132	0.0722	0.1306	0.4578	0.1253	0.1043

Supplementary Table 2: List of stabilizing salt bridges and hydrophobic interactions in HVEM-CD160 complex after molecular dynamics calculations

CHARACTER OF INTERACTION	PROTEINS	
	HVEM	CD160
salt bridges	Glu27	Arg89
	Arg75	Glu45
hydrophobic interactions	Pro17	Phe30
	Tyr23	Val32
	Thr33	Cys87
		Leu97
		Phe101
	Val36	Val20
		Cys87
		Arg89
		Leu97
	Cys37	Val32
